# Cross-border movement and women's health: how to capture the data

**DOI:** 10.1186/1475-9276-10-56

**Published:** 2011-11-21

**Authors:** Lisa Merry, Anita J Gagnon, Isabelle Hemlin, Heather Clarke, Jason Hickey

**Affiliations:** 1 School of Nursing, McGill University, 3506 University St., Montreal, QC, H3A 2A7, Canada; 2 McGill University Health Centre (MUHC), 2155 Guy St., Suite 400-7, Montreal, QC H3H 2R9, Canada; 3 Banque interrégionale d'interprètes, Agence de la santé et des services sociaux de Montréal, 4835 Christophe-Colomb Ave, 2nd floor Montreal, QC H2J 3G8, Canada; 4 Eastern Health, Waterford Bridge Road, St. John's, NL, A1E 4J8, Canada

**Keywords:** Questionnaires, Translations, Content validation, Emigration and Immigration, Women's Health

## Abstract

**Introduction:**

The movement of women across international borders is occurring at greater rates than ever before, yet the relationship between migration and women's health has been under-explored. One reason may be difficulty measuring migration variables including country of birth, length of time in country, immigration status, language ability, and ethnicity. A range of social, environmental, cultural, and medical characteristics associated with the pre-, during- and post-migration phases are also important to consider. The objective of this paper is to present challenges and solutions in measuring migration and related variables via survey-like questionnaires administered to international migrant women.

**Methods:**

The development, validation, and translation of two questionnaires subsequently applied in studies of migrant women during pregnancy, birth and postpartum were used as case examples to highlight related measurement issues.

**Results:**

Challenges: (1) Measuring socio-cultural, medical and environmental variables across the pre-during-post migration phases (since questions must be framed so that data relating to each phase of migration are captured); (2) Obtaining data for complex patterns of migration (i.e., multiple movements between multiple destinations); and (3) answering long questions across a time continuum.

Solutions: (1) Using interviewer-assisted rather than self-administered questions; (2) Adding probes and explanations to 'walk' participants through their migration experiences; (3) Identifying variables (e.g., trafficking) better captured using non-questionnaire data collection methods or better not collected (e.g., ethnicity) due to extreme variations in meaning.

**Conclusion:**

Carefully constructed and translated survey questionnaires are practical tools for the collection of a breadth of migrant data. These data, including detailed accounts of countries lived in, length of time in those countries, immigration status, change in status, language fluency, and health insurance eligibility offer rich descriptions of the population under study and make research findings with regards to migration more interpretable. Analyses by a range of migration indicators are facilitated through survey-like questionnaire data of this type.

## Introduction

Movement across international borders is a global phenomenon. The estimated number of international migrants in 2010 was 214 million of which 127 million were in industrialized regions, including Australia, Europe, Japan, New Zealand and North America [1]. Migrants include "immigrants", those who have moved to another country by choice for economic, education or family reasons [2]; "refugees", those who were forced to move due to war, civil unrest or other circumstances that threatened their survival [3]; "asylum-seekers", individuals who made refugee claims at a border or upon arrival in a country, and who in many instances are similar to refugees, i.e., were forced to flee their country for survival; "temporary workers", migrants who entered a country under specific employment programs; and "undocumented persons", those who have no status in the new country either because their temporary work or visitor status has lapsed or their refugee claim has been refused, or they decided to "immigrate" through irregular channels [2]. Migration experiences vary widely between groups and when examined together, migrants represent a range of ethnicities, cultures, lifestyles, religions, languages and experiences.

The health of migrants is a priority for many industrialized countries. In 2008 the World Health Assembly adopted a resolution calling on Member States to improve the health of migrants [4]. A Global Consultation on the health of migrants was held in 2010 to reach consensus on priority areas and strategies and to initiate an operational framework; monitoring migrant health was identified as a main priority. The development of migration indicators was recommended to ensure the standardization and comparability of data on migrant health and to support the appropriate aggregation and assembling of migrant health information [5].

Most health research with migrants to date is limited in the indicators used (e.g., only measuring country of birth). Studies also lack clarity in how migration variables are defined. A recent systematic review conducted to compare the rate of adverse perinatal outcomes between migrant and receiving-country-born women living in western industrialized countries could draw only limited conclusions because most studies defined their migrant population heterogeneously as "foreign-born"[6]. A key recommendation from this review was that researchers include more specific definitions of migrant participants and that they adjust for other migration risk factors in their analyses of effects on perinatal health. Descriptions of the operationalization definitions were also found to be lacking (e.g., many studies fail to specify how "refugee or immigrant status" was determined). One reason for the lack of migration data may be the difficulty in measuring migration variables. The development of tools and approaches to collect a range of migration-related variables would address the priority of developing migration indicators and improving data gathering to monitor migrant health.

The purpose of this article is to present challenges and solutions in measuring migration and related variables via survey-like questionnaires administered to international migrant women. Selected examples from two questionnaires are used as case examples to highlight related measurement issues.

## Background

### Migration factors

In our earlier work, we conducted two systematic reviews of the literature on refugee women's reproductive health [7,8]. The first review provides an extensive summary of factors deemed important in studying the health of this population [8]. Key migration factors identified include: country of origin, transit countries (and time in transit), ethnicity, rural or urban source area in country of origin, who made the decision to migrate (self or other), time spent in a refugee camp, arrival from an area of armed conflict, family separation, having been trafficked, immigration/legal status upon arrival, change(s) in immigration status, time spent in a detention centre, length of time in new country, relocations in new country, sense of belonging to a community, contact and connectedness to other countries (e.g., visits, internet, newspaper), employment, change in social status, acculturation, official language ability and access to services to learn language(s), access to interpreters, health insurance and access to health services, and availability of 'traditional' services.

The second review was conducted to identify existing questionnaires that could measure variables relevant to the health of refugee women resettling in new countries [7]. Several bio-psycho-social factors were considered including general health, torture, abuse, sex and gender-based violence, depression (including postpartum depression), stress, post-traumatic stress disorder, anxiety, somatization, social support, socio-economic status, discrimination, mother-child interaction as well as 'migration' and its related variables. Only five questionnaires were found to be relevant to migrant women in receiving countries. No tools for capturing migration data were identified.

### Social, environmental, cultural and medical factors

Gushulak & MacPherson (2004) [9] examined the role of movement on health and proposed a framework for describing the relationship between population mobility and health outcomes. They describe a range of factors involved in each phase (pre-, during- and post-migration) that influence health. *Pre-movement factors include: *levels of poverty, access to education, adequate housing, incidence of infectious and non-infectious diseases; environmental factors (e.g., weather, political, toxic); the status of health and social services including the availability, accessibility, and affordability of services; and cultural beliefs and practices regarding illness and health. The circumstances under which populations decide to migrate and how movement occurs also have major implications for health *during migration*. Migrants who are fleeing may be exposed to dangerous situations, such as lack of water, food, and shelter and may experience violence, unhygienic living conditions and unsafe modes of travel. Trauma, severe injury, and disease may result. *Post-migration*, migration status often determines access to services and shapes the integration process. Similar to the pre-movement phase, health status is also affected by the social, cultural, environmental and medical characteristics of the receiving country. One's capacity to adjust to the changes between their country of origin and the receiving country and 'integrate' into the new society is a key determinant of overall well-being.

### Migration to Canada

International migrants make up approximately 20% of the Canadian population [10]. The majority of migrants arrive as "immigrants", individuals who are evaluated and accepted on a point-system (e.g., for language skills, education) [11]. Canada also accepts and resettles convention refugees through the United Nations High Commissioner for Refugees (UNHCR) or accepted asylum claims within Canada [11]. Temporary migrants include "asylum-seekers", temporary workers and students [11]. All asylum claims are reviewed by an immigration board; accepted 'refugee claimants' may apply for residency status whereas rejected claimants are deported or remain in the country as "undocumented persons". Temporary workers and students must leave Canada by the end of the authorized period, though some remain and become "undocumented". The number of undocumented migrants is unknown.

Maintaining the health of migrants is a major public health concern in Canada. A recent Health Canada (2010) report that summarizes research on migrants highlights the World Health Assembly's 2008 resolution to improve the health of migrants and promotes an approach that includes action on the social determinants of health. The number and diversity of migrants and health disparities between different migrant groups suggests that migrant health research should attend to a range of factors to determine which are most important and for whom [12].

Using the information gleaned from the systematic reviews on women's reproductive health and related questionnaires, and Gushulak & MacPherson's (2004) framework for understanding the relationship between mobility and health, we developed, validated, and translated two tools capturing migration factors relevant to the reproductive health of international migrant women living in Canada. Both questionnaires were developed within a program of research on Migration and Reproductive Health over the span of six years. The Migration and Resettlement Questionnaire (MRQ) was developed first and sought to capture the range of variables identified in the systematic review while the Pre-During-Post Migration Questionnaire (PDPMQ) was developed later, and aimed to gather socio-cultural, medical and environmental data across the three migration phases described by Gushulak & MacPherson (2004). Both instruments were subsequently used in the context of Canadian studies examining the health and service needs of newly arrived migrant women during pregnancy, birth and postpartum [13,14]. Selected examples from each questionnaire are presented to highlight some of the challenges and solutions in measuring migration and related variables.

## Methods

### Cross-cultural instrument development, translation and validation

To successfully conduct research with migrants, a culturally adapted methodology and the use of well-translated tools must be exploited. A substantial amount of literature in the field of cross-cultural health research has been published [15-21]. While there is no agreed-upon best translation procedure, it is recognized that a rigorous process is necessary to optimize the quality of translated instruments and that the translation procedures must aim to attain cultural and linguistic equivalence between original and translated versions. Based on the translation literature, we devised a detailed plan for developing, translating and validating questionnaires for use with migrant women [7] (see Table 1 for a brief overview). This process guided the development of the MRQ and PDPQM.

**Table 1:** Steps for developing, translating and validating questionnaires for use with migrant women

1) Identification of the variables (based on the literature and input from migration/health experts)	Advisory committees consisting of healthcare professionals, representatives from non governmental organizations, and government officials all interested in migration and the reproductive health of migrant women are consulted
2) Identifying/drafting questionnaires	Questionnaires are identified by an extensive review of the literature

3) Assessing readability	Readability is assessed by counting the number of polysyllable words in each question^1^

4) Translation	To maximize the application of each translation to a broad population using the same language, translators with different backgrounds for each language are identified (e.g., Colombian and Mexican backgrounds for Spanish). The purpose and context of the studies in which the tool(s) will be administered are explained to translators Translators are instructed to use simple language and to avoid idioms and regional terms/expressions.^2^

5) Blind back translation	"Blind back-translation" is translation back into the source language by an independent translator unfamiliar with the original version of the questionnaire; Back-translated versions are compared to the original language version and discrepancies in wording noted and each item discussed and debated until agreement is reached on the optimal wording for the translated versions. When clarity is lacking in the original questionnaire, adjustments are made across all versions (original and translated).

6) Discussion groups with representatives from different ethno cultural communities	Migrant women representing a mix of ethno-cultural communities (Asia, South America, Africa, Europe) are asked to qualitatively assess the content validity and acceptability of the questions (i.e., feasibility to complete and cultural appropriateness); Groups generally consist of 5-10 participants

7) Administration of the translated questionnaires with monolingual individuals	Individuals who speak one of the 'translation languages' but not the original source language of the questionnaire, are asked to assess grammar, and ease of understanding of the translated version; They also assess the practical aspects of administration; 5-10 participants per language

8) Reliability testing (test retest and internal consistency as appropriate) of each language version as well as between the English and translated versions.	Reliability testing is completed via administration of the original and translated versions to persons fluent in both languages to ensure all language versions are understood in the same way and are equivalent

^1^National Literacy and Health Program: *Directory of plain language health information*. Ottawa: Canadian Public Health Association; 1999.

^2^Brislin RW: **The wording and translation of research instruments**. In *Field methods in cross-cultural research. Cross-cultural research and methodology series*. *Volume 8*. Edited by Lonner WJ, Berry JW. Thousand Oaks: Sage; 1986:137-164.

### MRQ

The MRQ was developed during a feasibility study meant to refine recruitment and data collection protocols, and to develop and test a battery of questionnaires for research with childbearing migrant women. Thirteen questionnaires, including the MRQ were validated and simultaneously translated from English into 12 languages based on the most recent refugee statistics for Canada at the time (since refugees are least likely to be able to speak English) [22,23]. Languages included: French, Spanish, Arabic, Tamil, Urdu, Chinese (complex and simplified characters), Serbo-Croatian, Farsi/Dari, Punjabi, Russian, and Somali.

An English version of the questionnaire was drafted and relevance of the selected migration factors (based on the systematic review) and corresponding questions were confirmed by the advisory committees working with our team. A draft version of the questionnaire was circulated for feedback. All recommendations were considered and made accordingly. Translation and back-translation were completed by the Inter-regional Interpreters Bank of the Health and Social Services Agency of Montreal. We sought to identify translators with different backgrounds for each language. Back-translations were reviewed during one meeting per language by LM, the Interpreter Services Agency person responsible for overseeing the translation work for our project (HC), and the two translators. Discrepancies in wording between the original and back-translated versions were noted and each item was discussed and debated until agreement was reached on the optimal wording for the translated versions. Where it was evident that clarity was lacking in the original questionnaire, adjustments were made to this version and changes were then incorporated accordingly across all translated versions.

To content validate and assess the acceptability of the MRQ for use with childbearing migrant women, the questionnaire was reviewed in ethno-cultural discussion groups and then tested with monolingual migrant women [24,25]. Reliability (bilingual) testing was begun but due to resources, feasibility issues in trying to complete the testing for several questionnaires across 13 languages (English and translations), and difficulties in finding enough respondents who fit our criteria, bilingual testing was not completed. The challenge of confirming psychometric properties across source and translated versions has been previously noted [16]. Subsequent use of the questionnaire in research studies provided additional information regarding the practical aspects of administration and the quality of responses obtained from individual questions.

### PDPMQ

Using Gushulak & MacPherson's (2004) framework, variables for this questionnaire were selected based on their relevance to migrant women. Specifically the PDPMQ aimed to capture: history/risk of exposure to infectious and non-infectious diseases, access barriers to health services, education, government funded services (monetary or other for food, child care, health care), language skills, conjugal social roles and responsibilities (e.g., whether a woman can work, has access to money, can make decisions about contraception, and can access health services without accompaniment or permission from a male family member); environmental factors such as type of job and risk for exposure to toxins or long hours; food security; injury experiences; and experiences of genital cutting (the latter because it is considered a cultural practice that can have significant repercussions in reproductive health). Questions were meant to obtain data for these variables for each of the three phases of movement, namely during the "time in their home country" (defined as the place where they lived the longest before age 12); during the "time between leaving their home country and arriving in Canada" (all destinations); and during their "time living in Canada".

A search for relevant literature/existing questions to measure the variables of interest was conducted and these were used to draft the initial version of the PDPMQ [26-33]. Similar to the MRQ, the PDPMQ was reviewed by experts in the field of migration and health and further refined. Because this tool was developed in the context of pilot-work, a limited budget prevented extensive translation and validation procedures. The instrument was however translated to French and Spanish, the two languages most relevant for our pilot study population. Translation was completed by two lay translators who were research assistants working within our program of research. The tool was then back-translated orally by two French/Spanish individuals recruited from the community who were unfamiliar with the questionnaire. Discrepancies were noted and changes were made immediately to the translated and original source version as needed. The revised version was reviewed by an ethno-cultural discussion group of migrant women living in Montreal, the final step to content validate this tool for use with a diverse population.

## Results

### Migration variables in the MRQ: Challenges and solutions in their measurement

A panel of clinicians, epidemiologists and health information experts across 22 countries recently identified five migration indicators that they considered the most important and feasible to measure for migration and health monitoring and research [34]. These included country of birth, length of time in receiving country, language fluency in receiving country language, immigration status and ethnicity [34]. These five variables were therefore chosen as examples to discuss the issues we faced in developing, translating, validating and using the MRQ.

#### Country of birth

The purpose of obtaining data on country of birth is twofold, the first is that it is useful in determining who is a migrant; the second is that it is the starting point of a migrant's trajectory and thus provides information on potential risk and protective factors to which a migrant might have been exposed. The question itself is straightforward, easily translated and relatively easily understood. We used this question as a base criterion in our studies to differentiate participants as migrants vs. non-migrants and with the exception of two infrequent occurrences it was useful in this regard. The two situations were: (1) Canadian citizens who had been born abroad and returned to live in Canada during childhood and have since lived in Canada, and (2) individuals born in Canada, who lived abroad for most of their lives and 'immigrated' back to Canada as adults. In the first instance it may be reasonable to categorize these individuals as non-migrants while in the second case it may be reasonable to categorize these individuals as migrants.

A second and more important issue with the country of birth variable is that it is relatively uninformative regarding exposure to risk (e.g., potential exposure to disease) and protective factors (e.g., preventive health measures such as immunizations) without knowing when and how long one lived in that country. One might be born in a country but may never have actually 'lived' there. Based on the literature, we considered asking about country of 'origin' or 'home country' as alternatives, defined either as the country one lived in the longest or as the place one considers their 'home/origin'. However, 'home' and 'origin' are open to being interpreted differently and would further complicate determining who was a migrant. Since we also wanted data on countries of transit, we formulated one question that asked participants to list in sequence all of the countries where they had lived and when (to the extent they could remember). While this question addressed the aforementioned issues, the question was long and was viewed as cumbersome to complete. Remembering dates was problematic and which countries were considered countries 'lived in' was sometimes unclear to participants. An additional change was made to the question to ask for the approximate number of years lived in each country rather than to dates. Completing the question via self-administration remained an issue however. Monolingual testing revealed that for many migrant women the concept of research itself was a foreign idea and completing questionnaires, an unfamiliar task. Interview administration and 'walking' participants through each question (i.e., "after you lived in that country, where did you live"?) was therefore required to obtain complete information.

#### Length of time in country

The importance of length of time in country is evident in the determination of who is a migrant (as described above). It is also understood that an individual who migrated 'long ago' is more familiar with and may be more similar to receiving-country-born individuals, compared to those recently arrived. Unsurprisingly, length of time in country was recommended by experts as the second most important migration indicator to capture [34]. While uncomplicated to measure, two issues merit consideration. The first is a lack of clarity on how to answer this question if an individual has moved in and out of the country (and related, how one is to respond to questions referring to the time when they arrived such as ('Did you arrive with family members?'). The second difficulty is memory recall regarding dates of movement.

The least complicated approach to gather these data on the MRQ was to combine length of time questions with those of country of birth and transit countries. To address the issue of how to respond to questions that refer to the time when they arrived in the new country, respondents were instructed to complete the questionnaire based on the *first *time they came to the country with the intention of staying for more than just a visit. These instructions were included on the questionnaire and were translated.

#### Language fluency

Language skills are challenging to assess through self-report on a questionnaire because one's own assessment of language abilities may be inconsistent with a more objective measure. Moreover, asking about language ability upon arrival in the new country (the variable that was of interest for the MRQ in order to know what language barriers they might have faced since their arrival) requires making an assessment about their ability in the past, which may have been many years ago and therefore, can result in inaccurate responses. MRQ questions developed to evaluate the self-assessment of language skills (reading, writing and speaking) in English and French upon arrival to Canada were based on questions used by Statistics Canada to evaluate language skills among immigrants [35]. These questions asked "How well did you know English/French when you first came to Canada?". A check-box format was used to assess their speaking, reading and writing abilities (i.e., fluently, well, with difficulty, not at all). Interview administration was necessary given the check-box format, which was difficult for participants who were unfamiliar with completing questionnaires.

In the PDPMQ (discussed below) we used different self-report questions to further assess language ability, also from a Statistics Canada census survey [28]. These included asking "Which language(s) do you know well enough to have a conversation?" "What language(s) do you speak most often at home" and "What is the language(s) that you first learned at home in childhood?" Despite their limitations, self report questions did provide a rapid (albeit crude) measure of language ability to assess for potential communication barriers in accessing services.

#### Immigration status

Obtaining information on immigration status is an extremely challenging undertaking, although key for knowing what health coverage and rights an individual has in the receiving country. Of utmost importance to migrants answering this question are its potential legal implications for them. Fear of jeopardizing an asylum application or fear of being reported to authorities can inhibit the sharing of immigration status or result in reporting false information. In the context of research with childbearing women, the challenge we largely faced was that in many instances women simply did not know their status because their husband or another family member had completed the immigration application on their behalf. For those who thought they knew their status, confusion often remained. For example, many asylum seekers would report being refugees. And those who reported being 'immigrants' were not always clear on whether they had come through family reunification mechanisms or as an economic immigrant with their spouse. Terminology also caused confusion. Some individuals were only familiar with the official immigration terms while others were not. These terms also did not easily translate across languages and therefore on translated versions of the questionnaire the immigration labels were not always well understood. Sometimes only the English/French terms were known even when a participant did not speak these languages. Finally, capturing changes in status added an extra layer of complexity due to memory recall, difficulties in knowing the various statuses they had had, and unwieldiness of the question (i.e., having multiple boxes to check and dates to complete).

We used a number of strategies to assist in obtaining data on immigration status. The first was to explain why these questions were being asked and to reassure respondents that all information would remain confidential. We listed the most common migration categories using official immigration terms; on some versions, English terms were put in brackets beside translated terms when translators recommended we do so. 'Other' was an option that could be checked with a line provided so that specifics could be added. Questions were limited to asking about status upon arrival and current status if they reported having changed status. In addition to these questions, the questionnaire also included a question on whether they had 'landed' (had residency status) upon arrival, whether they were sponsored to come to Canada (to determine if they came through family reunification), and if sponsored, who sponsored them, as well as a question about whether they had obtained citizenship. These additional data helped confirm the 'migration group' and provided further contextual information on the migrant's situation (e.g., a woman sponsored by her husband could give insight on power dynamics within the couple or her access to services). Rather than asking for specific dates, questions asked how long they had had the current status.

The questions were interview-administered and probes were used to help elicit responses. For example, for respondents who reported being refugees, probes added might be "Do you know yet if you will be allowed to stay in Canada?" "Have you had your hearing yet?". If they responded as not knowing, or offered any response related to timing of a hearing, we knew they were, in fact, not refugees but rather asylum seekers. Asking husbands/family members to assist in answering these questions (with consent of the participant) also facilitated their completion.

There were a number of limitations with the "immigration status" questions. For example, it was not possible to capture in a few straightforward questions whether a woman had been trafficked. Moreover, because the details on all statuses held were not collected, it sometimes caused uncertainty on how to categorize a participant (i.e., refugee, vs. asylum-seeker vs. non-refugee immigrant). Categorization rules were required due to the complexity of migration paths. For example, a woman might have arrived as an asylum seeker, and subsequently obtained permanent resident status through marriage. Or, a woman might have arrived through family reunification but with a refugee history. Therefore all data collected (length of time in country, country of birth, language upon arrival, and health insurance) had to be examined together to make an informed decision about her immigration class. Data on health insurance (i.e., in Canada, asylum seekers and refugees have access to a specific federal health insurance program) could differentiate refugees and asylum-seekers from non-refugees.

#### Ethnicity

Initially we developed an open ended question that simply asked respondents, "What is your ethnicity?". We then decided a close-ended question might facilitate answering the question, and so response options (e.g., African, Middle-Eastern) were added. When the question was reviewed by our ethno-cultural discussion groups the feedback was that "ethnicity" as a concept would not be understood. It was also felt that the response options represented geographic areas more than "ethnicities". When the question was piloted, many women left it blank. The literature defines ethnicity as a 'flexible', open and self-defined concept and includes the notion of people being bound by place, and by language, cultural or religious traditions, and lifestyles [36]. Capturing this range of possibilities for one variable within a larger group of questions was not feasible hence, the 'ethnicity' question was removed from the questionnaire.

#### General considerations

Table 2 provides examples of changes made to the MRQ through the translation and validation steps. Developing and translating across a number of languages with the aim of the tools being relevant to a cross-cultural population posed specific challenges. Despite using translators from different backgrounds (per language) it was difficult to avoid regional expressions/idioms particularly for languages spoken in many different countries around the world or languages with several dialects (e.g., Arabic, Punjabi). Similarly, keeping the level of vocabulary simple but avoiding colloquial language was a challenge. Verb tenses were difficult to capture in certain languages (e.g., Chinese) and as mentioned previously official immigration terms did not exist in most languages. For the ethnicity question terms for the various response options did not exist across languages (e.g., in Urdu there is no word for "Hispanic").

**Table 2:** Examples of major revisions of the MRQ based on a range of input

Original Questions	Input from key players	Input through «blind-back-translation»	Input from «ethno-cultural liaison groups (ECLGs)»	Input through «non-response rates from initial administration & additional feedback from key players»	Final Questions
**COUNTRY OF BIRTH/LENGTH OF TIME IN CANADA**					

-In which country were you born? -What date did you leave your country of birth? -Was Canada the first country you came to after leaving your own country? -If no, to what other countries did you go? -When did you arrive in Canada? - Did you spend any time living in one or more refugee camps?	-To fully capture the sequence and content of the migration experience a more detailed question on the number and name of countries where women lived was important to add. To know in which countries women spent time in camps was also important. **New question:**-To the best of your recollection, list in order all the countries you have ever lived in (including Canada) and when: a _______country of birth, ______month _ _ _ _ year to ______month _ _ _ _ year. Were you in a refugee camp? Yes No ** The question asked women to list every country beginning with the country of birth. There were 12 response spaces (i.e. a to l).	-"refugee camp" didn't translate correctly in some languages (e.g., the Dari back translation of refugee camp was "immigration camp").	ECLG suggested: -the question appeared too long (i.e. too many spaces to fill in). - women would not remember dates - the question about camp experience should be asked further down the questionnaire, grouped with other sensitive questions and should be preceded by a statement explaining why we are asking the information.	-Women had difficulty remembering specific dates and consequently did not complete the information. -Many women simply did not answer the question. - Further simplifying of the wording was suggested ("As much as you can remember" vs. "To the best of your recollection").	-As much as you can remember, **list in order **all the countries in which you have ever lived (including Canada) and for how long: a ____________, _____ years. country ** The list was reduced to five response spaces. -The questions on spending time in camp were moved and grouped with other questions on camp experiences, a question on time spent in a detention center and a question on whether time was spent in a country in armed conflict. An introductory statement was added: ***... ***"*These questions may be upsetting, but will help us further understand your migration experience and how it has affected your health*...

**IMMIGRATION CLASS**					

-Under which category did you come to Canada? ❑ Refugee ❑ Refugee claimant ❑ Immigrant ❑ Minister's permit ❑ Student ❑ Other _______ ❑ Husband sponsored ❑ Other ______	-Citizenship and Immigration Canada (CIC) provided more detailed information on the different immigration categories that exist. -To capture changes in immigration statuses an additional question was added. -There was interest to have migration information on the father of the baby as well. Same questions asked to the mother were added in a section of questions on the father. These additional data were also thought to be helpful to confirm the woman's status. **New questions: ***When completing the rest of the questionnaire please answer in relation to the first time you came to Canada with the intention of staying for more than just a visit*. 1- Did you come to Canada as a: ○ Temporary resident ○ Permanent resident ○ Refugee claimant 2- What was your immigration status? ○ Refugee (government sponsored) ○ Refugee (privately sponsored) ○ Refugee claimant/asylum seeker ○ Post-determination refugee ○ Independent immigrant (permanent resident/landed status) ○ Live-in caregiver ○ Student visa ○ Temporary worker ○ Provincial nominee ○ Minister's permit ○ Other specify_____________ ** An extensive list was provided so women could choose the one that applies to them. 3- Please read the following list and complete. Was your status ever: a Refugee (government sponsored) Yes No ___month _ _ _ _ year to ___ month _ _ _ _ year ** An extensive list of possible immigration statuses was provided.	-Translators thought that some women might have difficulty understanding the official CIC terms. -"Provincial nominee" was found to be a confusing term. Given the translators' feedback and that it is rare for women to come under this category this term was removed from the list ('other' was still left as an option so women under this category could write it in if it applied to them). -Similarly, "post-determination refugee" was thought to be a confusing term and is uncommon so was removed from the questionnaire. -"Live-in caregiver" didn't translate easily in certain languages (e.g., Somali back-translation was "dependent"; the Punjabi back-translation was "care-taker"; the Dari back-translation was "helper/volunteer"; in Russian the back-translation was "family educator"). Since this is a common migration category, it was kept and the best translation was determined together with the translators.	ECLG suggested: - questions were repetitive and too long - questions were asking too much detailed information (i.e. dates) -women may be reluctant to provide status information - felt that women would need to be reassured before answering these questions -suggested adding an introductory statement that would reassure women that information would remain confidential - -felt that the immigration categories were difficult to understand	- Women reported that the questions were repetitive and lengthy. Some women answered the first status question but not the subsequent questions. - Women needed assistance to complete the immigration status questions correctly [e.g. answered they came as refugee claimant in first question then responded they came as refugee (government sponsored) in second question]. - Many women left the "Was your status ever: " question incomplete. -Dates were also often left blank.	**- **To ensure women have the needed time and assistance to complete the questionnaire it will be administered in a home visit with a well-trained interviewer. - An introductory statement was added to the beginning of the questionnaire explaining why the information is being collected and reassuring women that the information will remain confidential and will not be passed on to CIC. -Question: "What was your immigration status?" was removed. - The list of immigrant categories was reduced even further (e.g., Minister's permit was removed). **Final questions: **-Did you come to Canada as a (please check one): ○ Immigrant (permanent resident/landed status) ○ Refugee ○ Refugee claimant/Asylum seeker ○ Temporary worker ○ Student ○ Visitor in Canada (no status) ○ Live-in caregiver ○ Other, specify_____________ -Has your immigration status changed since you arrived in Canada? Yes No -If your immigration status changed, what is your current status? (please check one) ○ Immigrant (permanent resident/landed status) ○ Refugee ○ Refugee claimant/Asylum seeker ○ Temporary worker ○ Student ○ Visitor in Canada (no status) ○ Live-in caregiver ○ Canadian citizen ○ Other, specify ____________ -How long have you had this status? ______________ (number of years)

**ETHNICITY**					

"What is your ethnicity?" _____	-Originally the question was grouped with the migration questions. The question was subsequently moved to the General Information questionnaire so that ethnicity data would be collected for all women, not just migrant women, -Response options were added to facilitate answering the question: ○ African ○ African-Canadian (American) ○ Asian & Pacific ○ Asian- Canadian (American) ○ Eastern-European ○ Western-European ○ Scandinavian ○ European-Canadian (American) ○ Jewish ○ Latin-American ○ Hispanic ○ Middle Eastern ○ Native Canadian (American) ○ Canadian ○ Other_________	-Suggested removing "(American)" -Suggested adding "Caribbean" as an option -Term "ethnicity" wasn't understood and came back in the back-translation as: race, nationality, ethnic belonging, ethnic origin -Generally there was difficulty across all languages to translate these terms: e.g., Native Canadian was understood as "born in Canada" rather than being understood as referring to "Aboriginal people" - Some of the terms didn't exist in certain languages e.g. There is no word for "Hispanic" in Urdu	-"I am not sure how to interpret this. I am not sure what I would put myself in the responses." -Women didn't understand what was really meant by "ethnicity" & were confused by the response options because they felt they represented geographical areas rather than ethnicity -They had difficulty answering the question themselves.	-Before the questionnaire was used in the study the response options were removed & the question was left open-ended -Women in the study didn't understand the question & many women left it blank	**-**The question was removed.

With respect to the translation process, back-translation was very useful for identifying discrepancies in what was meant in a given question versus what was actually understood by the translator. It was also helpful for finding translation errors, however meeting with both translators to review and discuss the translations/back-translations was excessively time consuming and sometimes translators felt pitted one against another creating an air of defensiveness in the discussions. Some practical challenges included working with multiple translators who varied in skill level since formally trained translators were not available for all languages. Translators' computer abilities also varied and formatting the questionnaire was therefore an issue for some, especially when questions had multiple response options or spaces to complete. Certain languages also took a lot of time to type because the languages have several more characters than there are keys on a standard computer keyboard (e.g., Chinese). This made it challenging to incorporate changes into these language versions. Software incorporating the fonts of some languages (e.g., Urdu) was difficult to obtain and some fonts were distorted depending on the computer where the file was opened. PDF file formats rectified this problem but caused additional complications when modifications to the questionnaire were required.

Verifying that errors were corrected in the translated versions was not really possible except for checking that the number of questions and response options matched the number in the original questionnaire. Using Arabic numerals across all language versions to number questions made this verification easier (it also aided when it came to data entry). Working closely with the translators/translation agency was important for minimizing errors.

The complete development, translation and validation process for all 13 questionnaires took over three years and required a coordinator dedicated to this endeavor. In addition to in-kind support provided from the Interpreter Services Agency, who acted as the coordinating body for the translation and back-translation process, the total cost approximated $300K CDN. The resources and time remained insufficient to fully accomplish what was planned (i.e., bilingual testing was incomplete). Therefore the heavy use of resources and time associated with translation and validation across several languages, and multiple instruments in this instance, is a major consideration, albeit not new, for researchers (and funding agencies) conducting migration and health research. An extensive translation and validation process does, however, optimize data quality, as suggested by Table 2.

### Migration variables in the PDPMQ: Challenges and solutions in their measurement

To highlight issues with the PDPMQ, four categories of variables are discussed: History/risk of exposure to infectious and non-infectious diseases; conjugal social roles and responsibilities; environmental factors and experiences of injuries. These variables presented greater challenges than some of the other variables (e.g., access barriers to health services, education, food security) which have been more commonly measured.

#### History/risk of exposure to infectious and non-infectious diseases

There are a number of challenges in gathering these data. (1) There is a long list of potential diseases that could be asked about, but not all are relevant to the different regions from which migrants may arrive. (2) Official medical terms may not always be known by participants, and lay labels vary by place and language. (3) Diagnoses may not have been given or diseases/infections may not have been diagnosed at all. (4) Stigma may be associated with certain illnesses, particularly STIs, making these additionally sensitive. (5) The most difficult challenge in collecting these data is that it was not always clear to the women when and where they had been exposed, making it difficult to answer in which movement phase they had had the disease/infection.

While it was impractical to include all possible lay terms or potential traditional diagnoses, interview administration permitted more explanations of the infections/diseases, with the use of lay terms if necessary, to try and determine if a participant had had any of these. For some illnesses, brief descriptions were also included on the questionnaire directly (e.g., for HPV, "warts around the vagina"). Memory triggers by interviewers assisted respondents to determine when they first had a disease/illness (e.g., "when did you first notice symptoms"). The final list was a 'short-list' of major infections and diseases, particularly health issues relevant to reproductive health, since our study population consisted of women of childbearing age. The list was compiled following reference to relevant sources [26,37] and input received through our network of migration-health researchers.

#### Conjugal social roles and responsibilities

Questions were drawn from the MEASURE DHS (Demographic Health Surveys), a project funded by the U.S. Agency for International Development [31]. This initiative supports national data collection through the use of surveys in more than 85 countries. The questions we used were from the "Women's Status Module" and included questions regarding marital status, whether the marriage was arranged, current living arrangement (i.e., with or without husband/partner), decision-making power (e.g., "Are important family decisions only made by the men of the family?") and attitudes regarding roles of men and women in the family (e.g., "If a woman is working outside the home should her husband help with housework?"). Questions about decision-making power and attitudes regarding women/men's roles questions were asked for each migration phase to assess women's statuses as well as changes in women's social functions and roles. Changes in attitudes and decision-making, being abstract, are not easily discernable as to when they may have occurred. Questions may also evoke feelings of being judged. Strategies to address these issues included using probes (e.g., asking women "Do you feel it's different here than when you were in your home country?") and reassuring women that there were no right or wrong answers.

#### Environmental factors

Questions for this section addressed risk of exposure to toxins in work environments and other factors that might indicate poor working conditions (e.g., long hours). Issues with these questions included respondents being unclear on how to respond if they had had more than one job or did not work for periods of time. Names of chemicals and toxins were unfamiliar to many and whether or not they were exposed to any of these was also often unknown. Respondents were asked to answer questions based on the employment they had had the longest. Examples of what or how they might have been exposed were specified in the questions (e.g., paints, animal bodies, gases, x-rays). Interview administration with probes to inquire about their places of employment also assisted participants in completing this section of the questionnaire.

#### Experiences of injuries

Risk of injury is greater for irregular migrants, i.e., those arriving through 'illegal' channels and those with a vulnerable status in the new country (asylum-seekers and undocumented persons) [9]. Injury-related questions included types of injuries experienced (e.g., broken bones), how the injuries occurred (e.g., fall), and where (e.g., school, work, home). Limitations of these questions included responses having been aggregated (i.e., all 'injury episodes' were asked about together to limit the number of questions) and the possibility that recalling these events may conjure traumatic memories for some. Response strategies included providing a definition of injury (i.e., "badly hurt"), adding an introductory statement to explain the purpose of collecting this information, and offering a referral to services for participants who become distressed.

#### General considerations

International migration research has largely used a 'primary migration model', which involves only two destination countries, the country of birth and the final destination, and assumes only one move, which is permanent. However globalization has resulted in patterns of migration that are complex and beyond the primary migration model. Other types of migratory flows which may involve multiple countries and/or frequent movement between destinations are on the rise [38]. While the PDPMQ aimed to go beyond the primary model of migration and to capture multiple movements, some simplification of the three movement phases was necessary to make the questions feasible for administration. The three phases of movement were therefore operationalized as follows: "time in home country" (rather than country of birth since they may not have lived very long in the country they were born), "time between leaving their home country to arrival in the receiving country" and "time since arrival in Canada". This approach however still assumes a unidirectional form of movement and although it captures more than one destination and participants are asked to provide a list of all countries lived in, the conditions and characteristics for each individual 'in-between destination' cannot be teased out.

Seeking data for three different time periods made structuring the questions a particular challenge for this questionnaire. In an effort to keep the questionnaire as brief as possible, as well as the amount of text that would need to be translated, the initial version was created by offering response options for each migration phase within a single question. Feedback from our ethno-cultural group was that this format was problematic. The questionnaire was then re-organized to have one form which contained questions referring to the "time in home country" and the "time in Canada" only. A separate form was created for the "during migration" questions and this form was to be administered only if a participant had transited through other countries. To help with recall and to minimize confusion, the migration phase(s) to which the question pertained was reiterated in each question. During administration, interviewers were also instructed to ask the questions referring to the two phases (home and in Canada) as though they were separate questions. Similar to the MRQ, interview administration and probes to help with memory recall, were necessary. In addition to format, careful attention to verb tenses (e.g., did/do; had/have) was also required to ensure relevancy of each question to the pre-and-post-migration phases.

## Recommendations and discussion

Survey-questionnaires, like the MRQ and PDPMQ offer the possibility of estimating a range of migration-related indicators for populations of migrant women. While migration data are thought to be a collection of more factual-type data, our results suggest that these data are not straightforward to obtain or to interpret.

### Interview-administer questionnaires and use probes and explanations

While it is generally recommended that questionnaires be self-administered to reduce interviewer bias, the diversity among migrant women (language dialects, literacy/education levels, familiarity with questionnaires) and the complexity of certain questions of the MRQ and PDPMQ necessitated interviewer assistance. Probes served as memory triggers, 'walked' participants through their experiences and in doing so, enhanced accuracy of responses. Explanations served to ensure questions were understood and to minimize confusion. To 'standardize' data collection, interviewers need to be extensively trained. An overview of immigration terminology and various immigrant classes, as well as examples of common and more complex immigration situations interviewers may encounter and guidance on how to approach complex cases, are imperative. A thorough review of all questions and challenges that may arise and where probes and explanations would be appropriate, and examples of probes and explanations to use, need to be provided. Consistency is also maximized by maintaining the same interviewers over the life a study and having ongoing discussions with them throughout the data collection period. Asking the interviewers to make notes when responses or cases are unclear can also aid in the interpretation of data collected.

### Identify difficult variables to measure

There are numerous migration variables that can be measured. Select variables from the MRQ and PDPMQ were discussed here, chosen based on recommendations from experts in the field or for the distinct measurement challenges they posed. Issues included some concepts being difficult to measure because they were more abstract, relative to one's circumstances and subjective (e.g., changes in conjugal social roles). Other questions were limited in capturing specifics (e.g., multiple statuses). Certain variables of interest, no matter how they were phrased, were difficult for participants because they necessitated having the knowledge to respond (e.g., knowing their immigration status). Other questions were sensitive and had the potential to cause distress. For some variables (e.g., trafficking) qualitative methods might be the only solution for capturing these data. Finally, the use of ethnicity in migration and health research should be questioned. The Delphi survey on migration indicators [34] recommended using 'maternal parent's place of birth' to define this concept- although feedback from our discussion groups suggested a geographical definition was too limited. Although ethnicity is a commonly used indicator and might seem useful particularly in countries where 'migrant' status is partly determined by blood lines (i.e., *Jus Sanguinis*), or for gathering data on potential genetic effects on perinatal health, it does not appear to be a concept that is universally understood by migrants. Moreover, there is general consensus among academics that ethnic categories are mainly social constructions which are not bound by genetic or biological differences [39]. The subjective and constructed nature of the concept of ethnicity, the difficulties in using pre-determined categories or alternatively an open-ended question, to 'measure' this concept and the uncertainty in how to interpret findings using these data suggest this variable may be better not collected.

### Translation considerations

Many of the issues we experienced in translating our questionnaires are commonly known [40]. However, our challenges were amplified given the number of languages and diversity of our population. Oral back-translation, where the translator and blind back-translator met directly to review each question (as was done with the PDPMQ), proved to be a more efficient and equally effective approach than written back-translation. In addition to immediately discussing translation differences, agreed upon translation changes could be incorporated without delay. Other implications for conducting large scale translation work for a diverse, multi-lingual migrant population are the resources in cost and time associated with this process. An important limitation of our work was the insufficient resources to complete reliability testing. However, questionnaires translated to multiple languages and validated with migrants allow for the collection of migration information from a broad segment of the migrant population including more difficult to reach groups who are often excluded from quantitative studies.

### Future research: Measurement of factors across movement phases

The complexity of measuring migration-related data is largely due to the 'space' and 'time' elements of the migration phenomenon. This was most obvious in the PDPMQ which sought to capture a range of socio-cultural, environmental and medical variables across the three movement phases, as described by Gushulak & MacPherson (2004) [9]. The main challenge was to structure and frame questions in a form that was relevant to each space (country) and clearly express to which timeframe questions were referring. Compounding this issue was that many of the variables could not easily be answered across a time continuum (e.g., history of infectious disease). Also, only movement in one direction could be obtained and the specifics for each country in which women lived were too cumbersome to collect. The PDPMQ was developed in pilot form and in only three languages, further development and validation is therefore needed for this tool. This questionnaire however is the first instrument of which we are aware that attempts to measure a range of socio-cultural, environmental and medical factors using a 'space-time' lens. Edberg et. al 2011 [41] observed that the interaction of factors contributing to health disparities among refugee and immigrant populations is inadequately documented. They also propose a trajectory model that considers the "diachronic interaction of a range of ecological factors" for understanding and assessing health disparities in these communities but note the lack of instruments with which to do so. Further research is needed to improve and create tools (i.e., for men and non-childbearing health contexts) that can capture the dynamic processes involved in the migration trajectory.

## Conclusion

The health of migrants presents an important public health challenge for governments and societies. There are a number of migration and related factors potentially associated with health outcomes. Many countries and agencies are working to improve health services, reduce access barriers and to address existing health inequities for migrant populations. To do so, appropriate and standardized data are needed to properly inform health policies and program development. Carefully constructed and translated survey questionnaires are practical tools for the collection of a breadth of migrant data. These data, including detailed accounts of countries lived in, length of time in those countries, immigration status, change in status, language fluency, and health insurance eligibility offer rich descriptions of the population under study and make research findings with regards to migration more interpretable. Analyses by a range of migration indicators are facilitated through survey-like questionnaire data of this type.

## Competing interests

The authors declare that they have no competing interests.

## Authors' contributions

LM coordinated the development, translation and validation of the MRQ and PDPMQ and drafted the manuscript. AJG was the principal investigator of the studies in which the questionnaire development, translation, and validation work was carried out. AJG revised the manuscript for important intellectual content. IH and HC coordinated the translation of the MRQ. JH developed the PDPMQ. All authors read and approved the final manuscript.
